# Plant functional trait data and reflectance spectra for 22 palmiet wetland species

**DOI:** 10.1016/j.dib.2018.08.113

**Published:** 2018-08-30

**Authors:** Alanna J. Rebelo, Ben Somers, Karen J. Esler, Patrick Meire

**Affiliations:** a Ecosystem Management Research Group (ECOBE), Department of Biology, University of Antwerp, Universiteitsplein 1C, Wilrijk 2610, Belgium; b Department of Conservation Ecology and Entomology, Stellenbosch University, JS Marais Building, Victoria Street, 7600, Private Bag X01, Matieland, 7602 Stellenbosch, South Africa; c Division Forest, Nature & Landscape, KU Leuven, Celestijnenlaan 200E, 3001 Leuven, Belgium; d Centre for Invasion Biology (C.I.B), Stellenbosch, South Africa

## Abstract

We provide reflectance spectra for 22 South African palmiet wetland species collected in spring 2015 from three wetlands throughout the Cape Floristic Region. In addition, we provide summarized plant functional trait data, as well as supporting and meta-data. Reflectance spectra were collected with a portable ASD Fieldspec Pro using standard methods. The 14 plant functional traits were measured on 10 replicates of each species, following standard protocols. We provide tables detailing these standard methods, as well a table with hypotheses on how these 14 continuous traits, as well as an additional 9 categorical traits, may affect ecosystem service provision. In addition, tables are attached which detail which functional and spectral groups these species belong to, according to the data. Finally, we include a photographic plate of the species data are provide for. We make these data available in an effort to assist in research on the understanding of how traits affect ecosystem service provision in wetlands, and particularly of whether remote sensing can be used to map these traits in wetlands.

**Specifications Table**

**Table:** 

Subject area	Earth and environmental sciences
More specific subject area	Remote sensing and plant ecology
Type of data	Tables (x9), image (photographic plate)
How data was acquired	Spectra: portable ASD Fieldspec Pro (ASD Inc., Boulder, USA).
	Functional traits: field measurements, laboratory analyses
Data format	Spectra: excel spreadsheet
	Functional traits: tables
Experimental factors	Spectra: Processed to reflectance, interference in major water absorption bands removed
	Functional traits: summarized; including meta-data
Experimental features	We measured spectral signatures (20 replicates) and 14 functional traits of 22 dominant South African palmiet wetland species in three wetlands within the Cape Floristic Region of South Africa.
Data source location	Cape Floristic Region, South Africa
	Theewaterskloof: 33°57′40.32′′S, 19°10′10.00′′E
	Goukou: 34° 0′30.46′′S, 21°24′59.97′′E
	Kromme: 33°52′24.69′′S, 24° 2′24.13′′E
Data accessibility	Data are provided in this article
Related research article	Rebelo, A. J., Somers, B., Esler, K. J., and P. Meire. 2018. Can wetland plant functional groups be spectrally discriminated? *Remote Sensing of Environment.* In press.

**Value of the data**

• The reflectance spectra could be used to form spectral libraries for these South African wetland species, and used in future hyperspectral remote sensing exercises (e.g. spectral unmixing).
• These spectra could additionally be used with other traits collected for these species to take the analysis further.
• The trait summary data could be used to augment meta-analysis; or international wetland studies.

## 1 Data

**Fig. 1:**
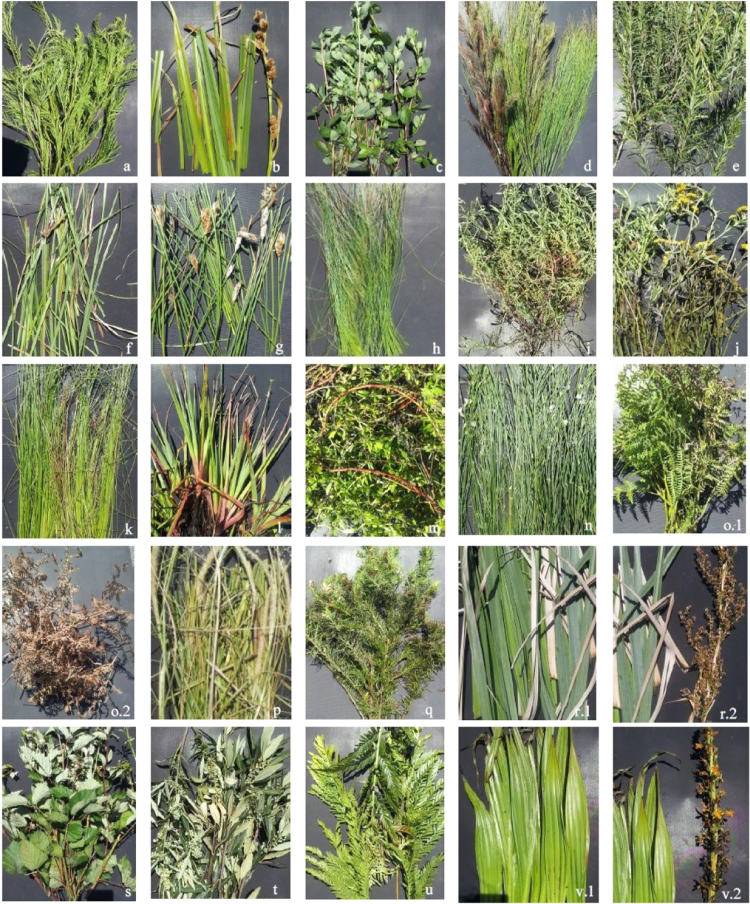
Photographs of the 22 dominant plant species in South African palmiet wetlands. The extra three photographs in this plate (*indicated by x.2*) are either of flowers or in the case of Bracken (*Pteridium aquilinum*), its characteristic dead form. The letters link the photographs to the species names in Table 3[1].

The dataset of this article provides reflectance spectra for wetland species as well as associated plant functional trait data [1]. The raw reflectance spectra for the 22 palmiet wetland species are included as an excel file (Appendix A). Meta-data about these measurements can be found in Table 1. Hypotheses about how each of the plant functional traits measured in this study may relate to ecosystem services is shown in Table 2. Table 3 gives details about the measurement (standard protocol) relating to each of the plant functional traits measured. Table 4 gives a summary of the data for each trait (for all 22 species). Table 5, Table 6 give additional output from analyses; the former simple regression analyses, the latter with partial least squares regression (PLSR). We performed PLSR using the ‘pls’ package [2] and ‘autopls’ code [3] in R to determine which PFTs could be predicted from the reflectance spectra. Table 7 details functional groupings of the 22 species and average trait values per group, whereas Table 8 does the same, but for spectral groups. Fig. 1 shows pictures of each of the 22 species.

**Table 1:** Species list of the 22 dominant plant species in South African palmiet wetlands and the wetlands they were recorded as being dominant in (from data recorded in plots) as well as the wetland the specimens for the reflectance measurements were collected from. Letters correspond to the photographs in Plate S1.

	**Species name**	**Growth form**	**Wetland dominant in**	**Wetland collected from**	**Number of spectra collected**
a	*Acacia mearnsii* (alien)	Tree	All	Goukou	20
b	*Carpha glomerata*	Graminoid	Theewaterskloof	Theewaterskloof	20
c	*Cliffortia odorata*	Shrub	Kromme	Somersetwest^a^	20
e	*Cliffortia strobilifera*	Shrub	All	Theewaterskloof	20
f	*Cyperus thunbergii*	Graminoid	Theewaterskloof, Kromme	Theewaterskloof	20
g	*Elegia asperiflora*	Graminoid	Goukou	Goukou	20
h	*Epischoenus gracilis*	Graminoid	Goukou	Goukou	16
i	*Helichrysum helianthimifolium*	Shrub	Goukou	Goukou	19
j	*Helichrysum odoratissimum*	Shrub	Kromme	Theewaterskloof	20
k	*Isolepis prolifera*	Graminoid	Theewaterskloof, Kromme	Theewaterskloof	20
l	*Juncus lomatophyllus*	Graminoid	Kromme	Theewaterskloof	20
m	*Laurembergia repens*	Annual	Theewaterskloof	Theewaterskloof	20
p	*Pennisetum macrourum*	Graminoid	Kromme	Theewaterskloof	20
r	*Prionium serratum*	Shrub	All	Theewaterskloof	20
n	*Psoralea aphylla*	Tree	Theewaterskloof	Theewaterskloof	20
q	*Psoralea pinnata*	Tree	Theewaterskloof	Theewaterskloof	20
o	*Pteridium aquilinum*	Shrub	Theewaterskloof	Theewaterskloof	20
d	*Restio paniculatus*	Graminoid	All	Theewaterskloof	20
s	*Rubus fruticosus* (alien)	Shrub	Theewaterskloof, Kromme	Theewaterskloof	20
t	*Searsia augustifolia*	Tree	Theewaterskloof	Theewaterskloof	20
u	*Todea barbara*	Shrub	Goukou	Goukou	20
v	*Wachendorfia thyrsiflora*	Forb	Theewaterskloof, Goukou	Theewaterskloof	20

a 34° 3′ 14.72′′ S; 18° 51′ 32.52′′ E

**Table 2:** Hypotheses of how the selected plant functional traits would be expected to link to Ecosystem Service provision (based on expert opinion). ↑ symbolizes a possible positive correlation, ↓ a negative correlation, → a non-directional relationship, and – signifies no relationship. *Italicized traits are categorical.*

**Table 3:** The 23 functional traits collected for the 22 species used in this study. All methods were based on the standardised protocol of Pérez-Harguindeguy et al. [4]. For categorical traits the codes assigned are shown in brackets.

	**Trait**	**Measurement method used**	**Unit**	**Scale**
Morphological/ Anatomical Traits	Shoot Length	Average shoot length of 10 mature plants	mm	Ratio
	Stem Diameter	Average diameter of 10 stems at base level	mm	Ratio
	Total Biomass	Average value of total biomass divided by number of mature shoots (in case of a tuft or rhizome)	g	Ratio
	Leaf Length/Width Ratio (LLWR)	Ratio between the length and the width of a leaf based on an average of 10 leaves	mm/mm	Ratio
	Leaf Dry Mass	Average leaf mass after being oven dried at 60 °C for 72 h (10 leaves)	mg	Ratio
	Leaf Area	Area of a single surface of a leaf based on an average of 10 leaves	mm^2^	Ratio
	Specific Leaf Area (SLA)	The total surface area of a leaf divided by its dry mass (based on an average of 10 leaves)	mm^2^/mg	Ratio
	Presence of Aerenchym	Scale of 1 to 3 (1 = no aerenchym, 2 = less than 50% aerenchym, 3 = predominantly aerenchym)	Class	Ordinal
	Woodiness of Stem	Scale of 1 to 3 (1 = no woody tissue, 2 = less than 50% woody tissue, 3 = predominantly woody tissue)	Class	Ordinal
	Hollowness of Stem	Scale of 1 to 3 (1 = stem not hollow, 2 = hollow space less than 50%, 3 = hollow space more than 50%)	Class	Ordinal
	Rooting Type	Adventitious (1), Taproot (2), Fine mesh (3), Annual (4), Tuft (tussock) (5), Rhizome (6), Stolon (7), Suffrutex (8)	Class	Nominal
	Growth Form	Geophyte (1), Forb (2), Annual (3), Graminoid (4), Shrub (5), Tree (6)	Class	Nominal
	Clonal Strategy	Tuft (1), Guerilla (2), Phalanx (3), Vegetative (4), None (0)	Class	Nominal
	Metabolism	C_3_(1), C_4_ (2), Parasitism (3), Carnivorous (4), CAM (5)	Class	Nominal
	Leaf Orientation	Plane (1), Stem (2), Base (3), Top (4), Leafless (0)	Class	Nominal
	Leaf Type	None (0), Simple -small narrow (1), Simple -larger round/narrow (2), Grass-like (3), Scale-like (4), Lobate (5), Palmate (6), Pinnate (7), Bipinnate (8), Pinnatifid (9), Long-leaf (10)	Class	Nominal
Biochemical Traits	Leaf C/N Ratio	Mass ratio of carbon versus nitrogen	g/g	Ratio
	Si Concentration	Biogenic silica was extracted from 25 mg dry plant (leaf and stem) material from 10 plants and analysed on an ICP	mg/kg	Ratio
	Si Content	Si concentration multiplied by average dry leaf mass to get an amount of Si per leaf	mg	Ratio
	Cellulose Concentration	Cellulose was measured by removing protein from 0.5–1 g of dry plant material from 10 plants, and by calculating mass before and after treatment with 72% sulfuric acid (Van Soest method)	%	Ratio
	Cellulose Content	Cellulose concentration (%) multiplied by average dry leaf mass to get an amount of cellulose per leaf	mg	Ratio
	Lignin Concentration	Lignin was measured by taking the results of the sulfuric acid digestion and weighing it before and after ashing at 550 °C (Van Soest method)	%	Ratio
	Lignin Content	Lignin concentration (%) multiplied by average dry leaf mass to get an amount of lignin per leaf	mg	Ratio

**Table 4:** Summary statistics for each of the continuous plant functional traits derived from 22 dominant plant species in South African palmiet wetlands.

	Plant Functional Trait	Mean	Min	Max	Median
Morphological/ Anatomical Traits	Shoot Length (mm)	1513.90	78.30	10500.00	1061.35
	Stem Diameter (mm)	38.76	0.13	450.00	11.13
	Total Biomass (g)	1280.86	0.20	15271.63	57.42
	Leaf Length/Width Ratio	12.97	0.00	88.40	2.80
	Leaf Dry Mass (mg)	2835.27	1.53	20430.00	146.14
	Leaf Area (mm^2^)	3420.28	31.70	16032.50	507.55
	Specific Leaf Area (SLA) (mm^2^/mg)	8.81	0.10	34.24	7.52
Biochemical Traits	Leaf C/N Ratio	42.71	16.61	85.86	40.29
	Si Concentration (mg/kg)	5045.75	80.00	31750.96	1328.03
	Si Content (mg)	7.99	0.00	87.03	0.37
	Cellulose Concentration (%)	29.60	15.67	44.91	29.01
	Cellulose Content (mg)	505.39	0.35	4165.15	39.80
	Lignin Concentration (%)	14.41	1.33	45.24	11.83
	Lignin Content (mg)	83.44	0.36	499.05	21.10

**Table 5:** The relationship between average reflectance over the four averaged sections of the spectrum and plant functional traits for five key traits. Both variables (average reflectance) and the plant functional trait were logged(10) in each regression.

Trait	Visible		NIR		SWIR		Total	
	Multiple *r*^2^	*p*-Value	Multiple *r*^2^	*p*-Value	Multiple r^2^	*p*-Value	Multiple *r*^2^	*p*-Value
Cellulose content (mg)	0.36	<0.01	**0.49**	**<0.01**	0.40	<0.01	0.46	<0.01
Lignin content (mg)	0.28	<0.05	**0.54**	**<0.01**	0.43	<0.01	0.49	<0.01
Si content (mg)	0.18	<0.05	0.22	<0.05	**0.30**	**<0.01**	0.29	<0.01
Leaf mass (mg)	0.16	NS	0.37	<0.01	0.36	<0.01	**0.38**	**<0.01**
Leaf area (mm^2^)	0.26	<0.05	0.36	<0.01	0.39	<0.01	**0.41**	**<0.01**

**Table 6:** Model performance parameters for partial least squares regression (PLSR) of predicting plant functional traits from reflectance spectra of 22 South African wetland species for four different parts of the spectrum: UV-A, visible, NIR and SWIR. Abbreviations: nlv is the number of latent variables, r2, the coefficient of determination, is given for model calibration and validation, as is RMSE: the root mean square error. Shaded cells show r2 (calibration) values of greater than 0.40.

**Table 7:** Functional groups of 22 dominant South African wetland species based on cluster analysis with 23 functional traits. The top 10 predictors (traits) driving the separation of groups are shown as average values per functional group. The numbers in brackets indicate the importance of each predictor in driving the grouping. For categorical traits the number given is not an average but the mode (most common form of the trait). Corresponding categories for these codes can be found in Table 3.

Species	Functional Group	Cellulose Content (1.00)	Leaf Area (0.90)	Leaf Orientation (0.54)	Leaf Type (0.50)	LLWR (0.42)	Lignin Content (0.37)	C/N Ratio (0.24)	Rooting Type (0.21)	Woodiness (0.21)	Clonal Strategy (0.20)
*Acacia mearnsii*	1	101.30	1453.76	4	1	3.23	98.01	24.33	2	3	0
*Cliffortia strobilifera*											
*Psoralea aphylla*											
*Psoralea pinnata*											
*Cliffortia odorata*	2	13.41	622.53	2	2	2.79	9.90	35.56	1	3	4
*Helichrysum helianthemifolium*											
*Helichrysum odoratissimum*											
*Laurembergia repens*											
*Rubus fruticosus*											
*Searsia augustifolia*											
*Pteridium aquilinum*	3	21.39	175.43	1	8	5.63	14.41	23.48	1	2	0
*Todea barbara*											
*Restio paniculatus*	4	61.47	1329.34	0	0	0.00	20.41	62.71	6	2	1
*Elegia asperiflora*											
*Epischoenus gracilis*											
*Isolepis prolifera*											
*Cyperus thunbergii*	5	174.84	4529.75	3	10	56.42	39.15	70.45	6	1	3
*Juncus lomatophyllus*											
*Pennisetum macrourum*											
*Carpha glomerata*	6	3273.22	15479.52	3	10	25.05	385.47	39.90	6	1	0
*Prionium serratum*											
*Wachendorfia thyrsiflora*

**Table 8:** Spectral groups of 22 dominant South African wetland species based on cluster analysis with 1678 individual reflectance spectra. The top 10 predictors (spectra) driving the separation of groups are shown as average values per spectral group. The numbers in brackets indicate the importance of each predictor in driving the grouping.

Species	Spectral Group	539 nm (1.00)	540 nm (1.00)	538 nm (1.00)	541 nm (1.00)	542 nm (1.00)	613 nm (1.00)	535 nm (1.00)	536 nm (1.00)	609 nm (1.00)	610 nm (1.00)
*Carpha glomerata*	1	6.05	6.13	5.96	6.21	6.27	6.06	5.68	5.78	6.09	6.08
*Cliffortia strobilifera*											
*Elegia asperiflora*											
*Epischoenus gracilis*											
*Helichrysum odoratissimum*											
*Juncus lomatophyllus*											
*Laurembergia repens*											
*Pteridium aquilinum*											
*Psoralea pinnata*											
*Acacia mearnsii*	2	7.33	7.45	7.21	7.55	7.64	6.72	6.81	6.95	6.77	6.76
*Cliffortia odorata*											
*Psoralea aphylla*											
*Rubus fruticosus*											
*Todea barbara*											
*Restio paniculatus*	3	6.16	6.24	6.07	6.32	6.4	6.52	5.8	5.89	6.53	6.52
*Helichrysum helianthemifolium*											
*Pennisetum macrourum*	4	12.92	13.07	12.76	13.2	13.33	14.61	12.26	12.42	14.59	14.6
*Prionium serratum*	5	13.75	13.94	13.54	14.1	14.25	12.46	12.89	13.11	12.59	12.56
*Wachendorfia thyrsiflora*											
*Cyperus thunbergii*	6	10.58	10.71	10.43	10.83	10.94	10.4	9.95	10.11	10.45	10.44
*Isolepis prolifera*											
*Searsia augustifolia*

## 2 Experimental design, materials, and methods

These data form part of the Supplementary material of a publication in Remote Sensing of Environment [1]. Relevant sections from the methods have been extracted from this publication.

### 2.1 Study design

Species composition data were obtained from 39 plots in the three different palmiet wetlands. Plots were arranged on seven transects (100–200 m) along cross sections through the wetlands, with six plots (3×3 m) placed between 20–50 m apart, yielding a total of 36 plots. In the Goukou wetland, three extra plots were added to fully capture variation in plant communities. Species and their relative abundances were recorded in each plot, using the Braun-Blanquet Scale [5]. Dominant species were defined as those making up more than 25% cover in any plot. The resultant 22 species are listed in Table 1, Fig. 1. Ten mature specimens from each dominant species were collected from their wetland of origin for measurement of PFTs at the respective field station or in the lab (depending on the trait). Traits were collected once for each species from random specimens in the field (maximum abundance approach, Carmona et al. [6]). Extra specimens were collected from one of the three sites for each species (Table 1).

### 2.2 Plant functional traits

We measured 23 PFTs, each selected as they were predicted to have a link to at least one wetland ecosystem service (Table 2). Definitions and methods for the measurements of each PFT are given in Table 3; and for all commonly used PFTs we used the standardized protocol for measurements [7]. Of the PFTs measured, 16 were morphological/anatomical, and seven were biochemical in nature (Table 3). For biochemical traits, samples were cleaned, dried at 70 °C for 48 h, ground and homogenised using a mill to 0.5 mm particles. Total carbon and total nitrogen were determined by total combustion of 5 mg of each sample on a Flash 2000 CN-analyzer (Thermo Fisher Scientific). To determine plant silicon content, we used a procedure for extracting biogenic silica (Schoelynck et al. 2010), which involved incubating a 25 mg sample of dried plant material in a 0.1 m Na_2_CO_3_ mixture which was placed in a water bath at 80 °C for 4 h. This dissolved biogenic silica was then spectrophotometrically analysed on a Thermo IRIS inductively coupled plasmaspectrophotometer (ICP; Thermo Fisher, Franklin, MA, USA). Plant lignin and cellulose content were measured using the Van Soest method [8]. Summary statistics are shown for each of the continuous PFTs in Table 4.

### 2.3 Reflectance measurements

Plant canopy spectra were measured in the field in November 2015 (spring) under clear sky conditions within two hours of local solar noon. Phenology has been shown to be valuable in discriminating wetland species (e.g. reed beds) and spring is the season in which interspecific phenological distinctions are generally at their greatest [9], [10]. All reflectance measurements were taken with a portable ASD Fieldspec Pro (ASD Inc., Boulder, USA). The probe was held at a constant distance of 60 cm above the surface (25° FOV; diameter 26.59 cm), keeping the sensor perpendicular to the angle of the sun. Live (wet) specimens from each species were arranged on a large matt black (non-reflective: uniform < 5% reflectance across the 350–2500 nm range) surface (1.5×2 m), with leaves facing upwards (adaxial surface up) where possible. This measurement set-up allowed us to measure the reflectance of individual plant species without background contamination originating from soil or other plant species. This set-up thus allowed us to make a one-on-one comparison between reflectance and PFTs. It is acknowledged that the spectral effects of 3D canopy structure (i.e. volume scattering effects) were not fully captured with this set-up. Since this study focussed primarily on leaf traits, this is not expected to present any problems.

Twenty spectral signatures were collected for each species. There were two cases where data had to be excluded due to equipment problems (see Table 1 for details). Between readings for each species, the ASD was optimised using a spectralon (Spectralon®, Labsphere, North Sutton, USA) and white reference measurements were captured. Spectra were collected over the range of 350–2500 nm with 1 nm intervals. ASD binary files were first converted to ASCII reflectance files using ViewSpecPro and subsequently post-processed to remove data in the water absorption bands at 1350–1460 nm and 1790–2000 nm as well as noise at 2350–2500 nm.
