# Supplementary material for: Plant functional trait data and reflectance spectra for 22 palmiet wetland species
Source: Data Brief. 2018 Aug 30;20:1209–19. doi: 10.1016/j.dib.2018.08.113 (PMC6143752; doi:10.1016/j.dib.2018.08.113)
Supplement: Supplementary file 1 — Supplementary material [file mmc1.docx]

Conflict of interest form

There was no conflict of interest form provided on the website, nor in the e-mail I received from the journal. Therefore I cannot complete the form until I receive it from DIB.
